# Closing the Loop: Unexamined Performance Trade-Offs of Integrating Direct Air Capture with (Bi)carbonate Electrolysis

**DOI:** 10.1021/acsenergylett.4c00807

**Published:** 2024-05-01

**Authors:** Hussain M. Almajed, Recep Kas, Paige Brimley, Allison M. Crow, Ana Somoza-Tornos, Bri-Mathias Hodge, Thomas E. Burdyny, Wilson A. Smith

**Affiliations:** † Department of Chemical and Biological Engineering, University of Colorado Boulder, Boulder, Colorado 80309, United States; ‡ Renewable and Sustainable Energy Institute, University of Colorado Boulder, Boulder, Colorado 80309, United States; § National Renewable Energy Laboratory, Golden, Colorado 80401, United States; ∥ Department of Chemical Engineering, Delft University of Technology, Van der Maasweg 9, 2629 HZ Delft, The Netherlands; ⊥ Department of Electrical, Computer and Energy Engineering, University of Colorado Boulder, Boulder, Colorado 80309, United States; # Department of Applied Mathematics, University of Colorado Boulder, Boulder, Colorado 80309, United States

## Abstract

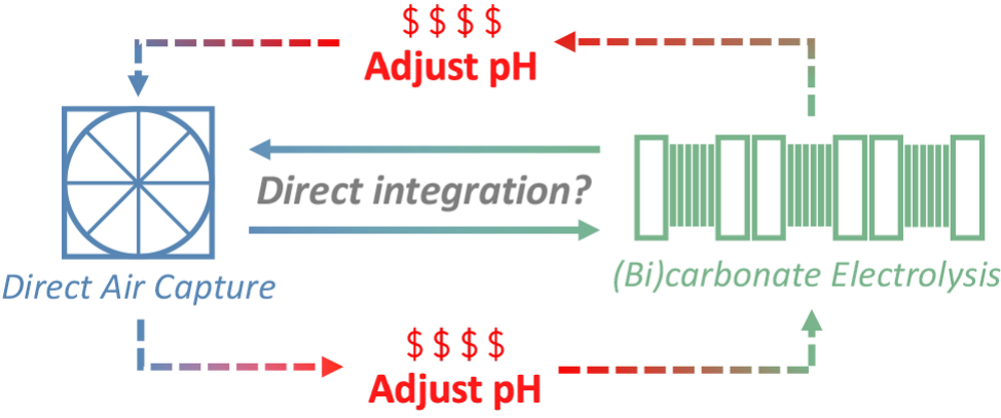

CO_2_ from carbonate-based capture solutions requires a substantial energy input. Replacing this step with (bi)carbonate electrolysis has been commonly proposed as an efficient alternative that coproduces CO/syngas. Here, we assess the feasibility of directly integrating air contactors with (bi)carbonate electrolyzers by leveraging process, multiphysics, microkinetic, and technoeconomic models. We show that the copresence of CO_3_^2–^ with HCO_3_^–^ in the contactor effluent greatly diminishes the electrolyzer performance and eventually results in a reduced CO_2_ capture fraction to ≤1%. Additionally, we estimate suitable effluents for (bi)carbonate electrolysis to require 5–14 times larger contactors than conventionally needed contactors, leading to unfavorable process economics. Notably, we show that the regeneration of the capture solvent inside (bi)carbonate electrolyzers is insufficient for CO_2_ recapture. Thus, we suggest process modifications that would allow this route to be operationally feasible. Overall, this work sheds light on the practical operation of integrated direct air capture with (bi)carbonate electrolysis.

Achieving global net-zero climate targets by the end of the century requires the capture of carbon dioxide (CO_2_), either in concentrated forms or directly from the atmosphere using CO_2_ removal (CDR) technologies.^1,2^ One of the most promising CDR pathways is via direct air capture (DAC), which uses a solid/liquid solvent (e.g., KOH) or sorbent (e.g., cellulosic-based amines) to capture CO_2_ from the atmosphere.^3−5^ Although both solid/liquid solvents and sorbents have been commonly used in DAC applications, the current most cost-effective and scalable option is the liquid alkaline solvent.^3^ In a typical liquid alkaline DAC process, ambient air passes through a CO_2_-absorbing medium in an air contactor, forming a CO_2_–adduct intermediate, which can be integrated into CO_2_ removal and solvent recycling techniques.^3^ These routes have enabled the foundation of rapidly developing DAC companies such as Climeworks,^6^ Carbon Engineering,^7^ and Global Thermostat.^8^

Unfortunately, the reported total energy consumption of DAC (i.e., CO_2_ capture and regeneration from air) is high, ranging from 5.50 to 9.50 GJ/t-CO_2_ (i.e., from 242.1 to 418.1 kJ/mol-CO_2_).^2^ Because CO_2_ capture is highly exothermic (eqs 1 and 4), its release from the capture solvent requires substantial regeneration energy to recover the captured CO_2_ in a high-purity form and allow for the solvent to be regenerated in order to recapture fresh CO_2_.^3,5,9,10^ Concentrated hydroxide-based DAC processes, which capture CO_2_ using hydroxides to form carbonates, requires a particularly high temperature (≥900 °C) to dissociate the metal carbonate into metal oxide and CO_2_ via calcination.^5,11^ This high temperature is challenging to reach via electrical energy input alone and requires a thermal energy input of 4.05 GJ/t-CO_2_ (i.e., 178.2 kJ/mol-CO_2_).^5,12,13^ Comparatively, the established monoethanolamine (MEA) solvent recovery method can be performed at much milder temperatures of 80–120 °C, which can be achieved from waste heat and renewable electricity integrations. However, the MEA/CO_2_ regeneration step still requires a regeneration energy input in the range of 2.00–5.50 GJ/t-CO_2_ (i.e., 88.0–242.1 kJ/mol-CO_2_).^14−16^

The costly energetics of recovering CO_2_ from capture solvents has motivated efforts to combine the CO_2_ removal step with a CO_2_ conversion step,^14,17−20^ effectively integrating CO_2_ capture and conversion into a single cycle. For the hydroxide route, for example, CO_2_ will leave the air contactors in the form of both bicarbonates and carbonates (hereinafter referred to as (bi)carbonates). Electrolyzers utilizing reverse-biased bipolar membranes (BPMs), which separate the cathode from the anode and split water into protons and hydroxides, can then modulate the pH of the (bi)carbonate solution to generate CO_2_*in situ*. The CO_2_ can then be further reduced into more valuable intermediates or products.^19^ By the absence of the energy-intensive CO_2_ regeneration steps, the thermodynamic favorability of directly converting captured CO_2_ to products would be very compelling.

Figure 1 qualitatively shows the energetics of the sequential and direct integration routes of CO_2_ capture and conversion, highlighting the required regeneration of CO_2_ in the sequential routes as opposed to the direct conversion of (bi)carbonates in the direct integration route. While the putative mechanism in both the sequential and direct pathways is the conversion of molecular CO_2_ (either fed directly or generated *in situ*), we choose to use the terminology “(bi)carbonate electrolysis” to distinguish between the CO_2_ sources and to be consistent with the phrasing in the literature.^10,17,18,20−27^ It is also worthwhile to note that CO_2_ electrolysis takes a feed of gaseous CO_2_ whereas (bi)carbonate electrolysis takes a feed of liquid (bi)carbonates, which enables the possibility of directly integrating CO_2_ capture and conversion. Thus, multiple research groups have proposed the replacement of the high-temperature (900 °C) solvent/CO_2_ regeneration steps in hydroxide-based DAC with a single low-temperature (≤80 °C) (bi)carbonate electrolysis step, aiming to concurrently regenerate the hydroxide-based solvent and produce more desired products such as CO or syngas (i.e., a mixture of CO and H_2_).^10,17−20,28,27,21^

**Figure 1:**
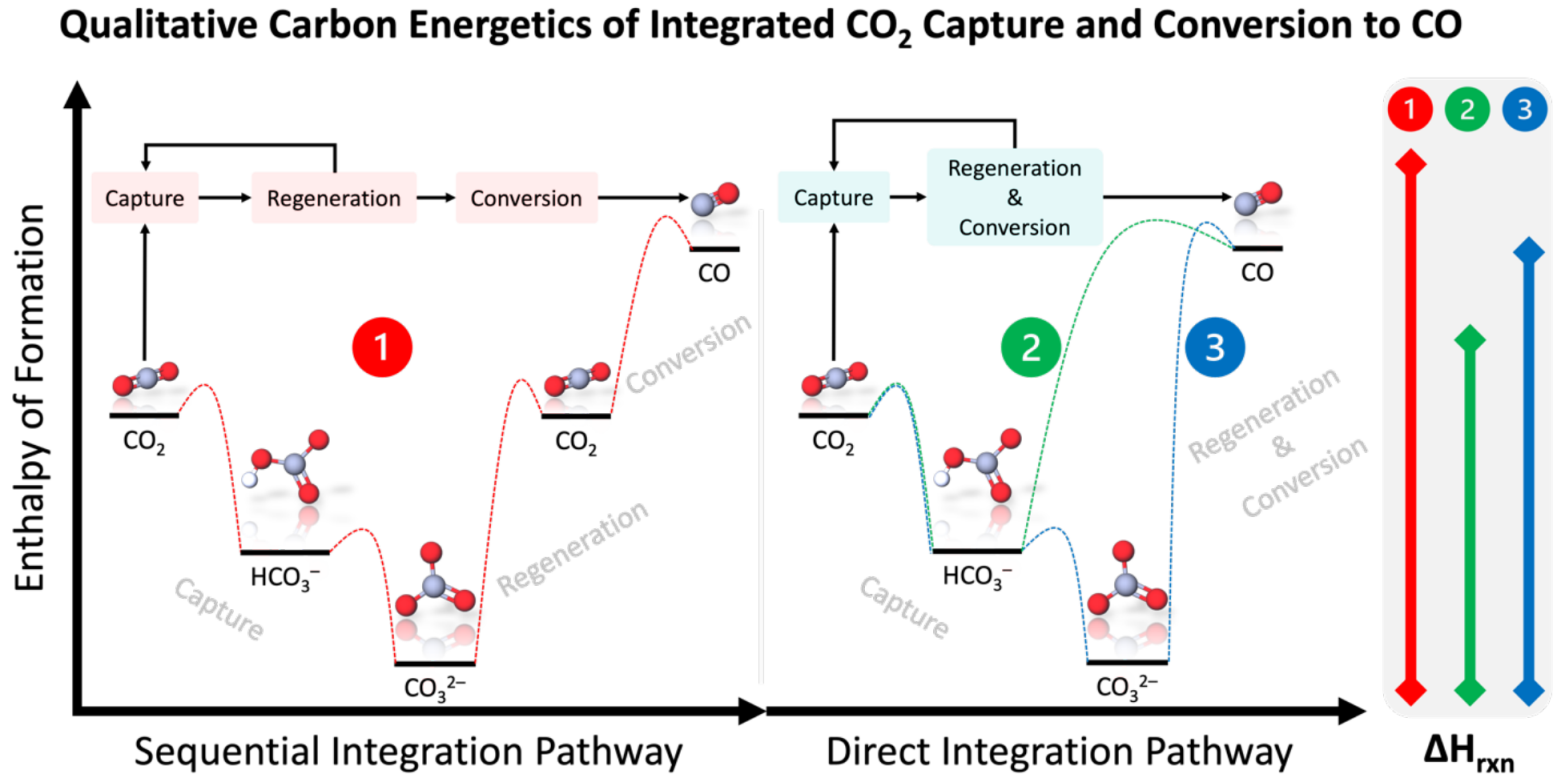
Qualitative carbon energetics of CO_2_ capture and conversion to CO via the sequential integration pathway (i.e., integrated air contactor, CO_2_ regeneration, and CO_2_ conversion: 1, red) and via the direct integration pathway (i.e., integrated air contactor with bicarbonate and carbonate conversions: 2 and 3, green and blue). Note that we do not show the detailed mechanism of these molecular transformations but indicate the presence of transition states in each. We do not include the in situ CO_2_ regeneration in the direct integration pathway to highlight the promise of combining the regeneration and conversion steps into a single step. Also note the qualitative energy requirement scale at the right-hand side of the figure, which qualitatively emphasizes the thermodynamic favorability of directly converting captured CO_2_ (i.e., (bi)carbonates) into desired products.

In the past few years, there have been many examples of integrated CO_2_ capture and (bi)carbonate electrolysis proposed in the literature. For example, Li et al.^20^ attempted to integrate the capture and conversion steps in a lab-scale system, where CO_2_ was captured by a 2 M KOH solution in a bottle and the captured (bi)carbonate mixture was converted to syngas in an electrolyzer with a BPM. The authors were able to show continuous syngas production at an H_2_:CO ratio between 2:1 (Faradaic efficiency of CO (FE_CO_) ≈ 33%) and 3:1 (FE_CO_ ≈ 25%) for 145 h at 3.8 V (energy efficiency (EE) ≈ 35%) and 200 mA/cm^2^.^20^ Later, Xiao et al.^27^ used the same setup but with a cation exchange membrane rather than a BPM and with a CO_2_ diffusion adlayer that limited the transfer of protons to the catalyst layer, improving the overall carbon efficiency of the process. With these changes, they were able to improve the FE_CO_ to 40% and reduce the cell voltage to approximately 3.3 V (EE ≈ 40%) at 100 mA/cm^2^, although their system was tested for only 23 h.

Lees and co-workers^21^ designed a bicarbonate electrolyzer with a BPM, intending to integrate it with an air contactor to develop an energy- and cost-efficient air-to-syngas/CO system. They utilized a 3 M KHCO_3_ catholyte and were able to achieve a FE_CO_ of 82% (H_2_:CO ratio ≈ 0.2) at a current density of 100 mA/cm^2^ and a cell voltage of 3.5 V (EE ≈ 38%). However, they found that a higher applied current density of 200 mA/cm^2^ not only increases the cell voltage but also reduces the FE_CO_ to about 60% (H_2_:CO ratio ≈ 0.67).^21^ Further work by Zhang et al.^22,23^ demonstrated that changing the anodic reaction to H_2_ oxidation and applying higher pressures of up to 4 atm can increase the FE_CO_ back to high levels (≥80%) and reduce the cell voltage to around 2.00 V (EE ≈ 67%), however at current densities of ≤100 mA/cm^2^. More recently, Kim et al.^18^ built an integrated system composed of a stainless steel CO_2_ absorber and a (bi)carbonate electrolyzer. They used a 1 M K_2_CO_3_ solution as the capture solvent to produce KHCO_3_, which could be fed to the electrolyzer for high CO formation and selectivity. Their integrated system was able to produce syngas at an H_2_:CO ratio of 1.5–2.3 (FE_CO_ ≈ 30–40%), a cell voltage of 3.5 V (EE ≈ 38%), and a current density of 100 mA/cm^2^.^18^

The contribution of these efforts, summarized in Table 1, has enabled the research field to understand and improve (bi)carbonate electrolysis of CO_2_, providing valuable insights into both the opportunities and limitations of the technology. A key missing piece of research to date, however, is a discussion on the trade-offs of a fully closed integrated capture-and-conversion loop (Figure 2a). Most critically, a circular CO_2_ capture-and-conversion process requires the outlet solvent of a (bi)carbonate electrolyzer to recapture CO_2_ again once passed through an air contactor. For this to be possible, the (bi)carbonate electrolyzer must subsequently release and convert most of the absorbed CO_2_. As an example, if an air contactor captures CO_2_ with a 1.00 M KOH solvent, a (bi)carbonate electrolyzer should be able to return the same 1.00 M KOH back to the air contactor. To the best of our knowledge, the ability of the catholyte outlet to recapture CO_2_ continuously for long durations (≥1000 h) has not yet been demonstrated experimentally, which is an essential step for providing long-term, large-scale, and durable CDR.

**Table 1:**
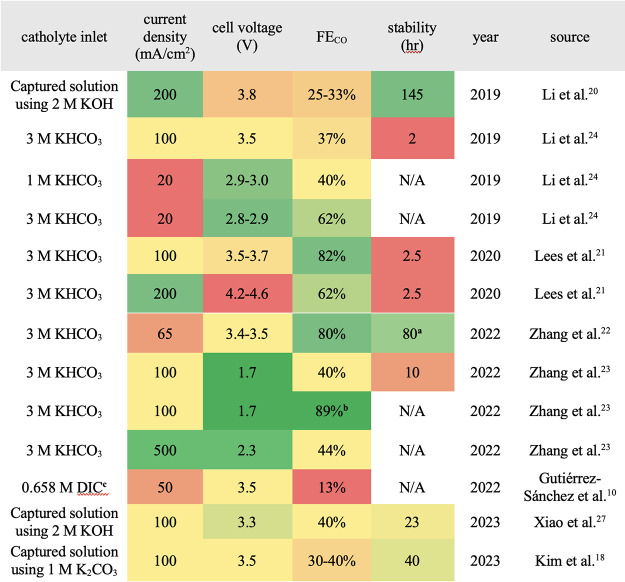
Summary of Previous (Bi)carbonate Electrolysis Works That Considered the Direct Integration Route^d^

a The stability test was run for 80 h with KHCO_3_ being refreshed every 500 s.^22^

b Pressurized to 3.5 atm to yield 89%, but no stability/durability test was provided.^23^

c DIC: dissolved inorganic carbon. The 0.658 M DIC contains 0.166 M HCO_3_^–^ and 0.492 M CO_3_^2–^.^10^

d The performance metrics (columns) are separately color-coded. Greener colors show the best performance metric achieved, and the redder colors show the worst performance metric achieved. N/A results were not provided by the authors of the cited works.

**Figure 2:**
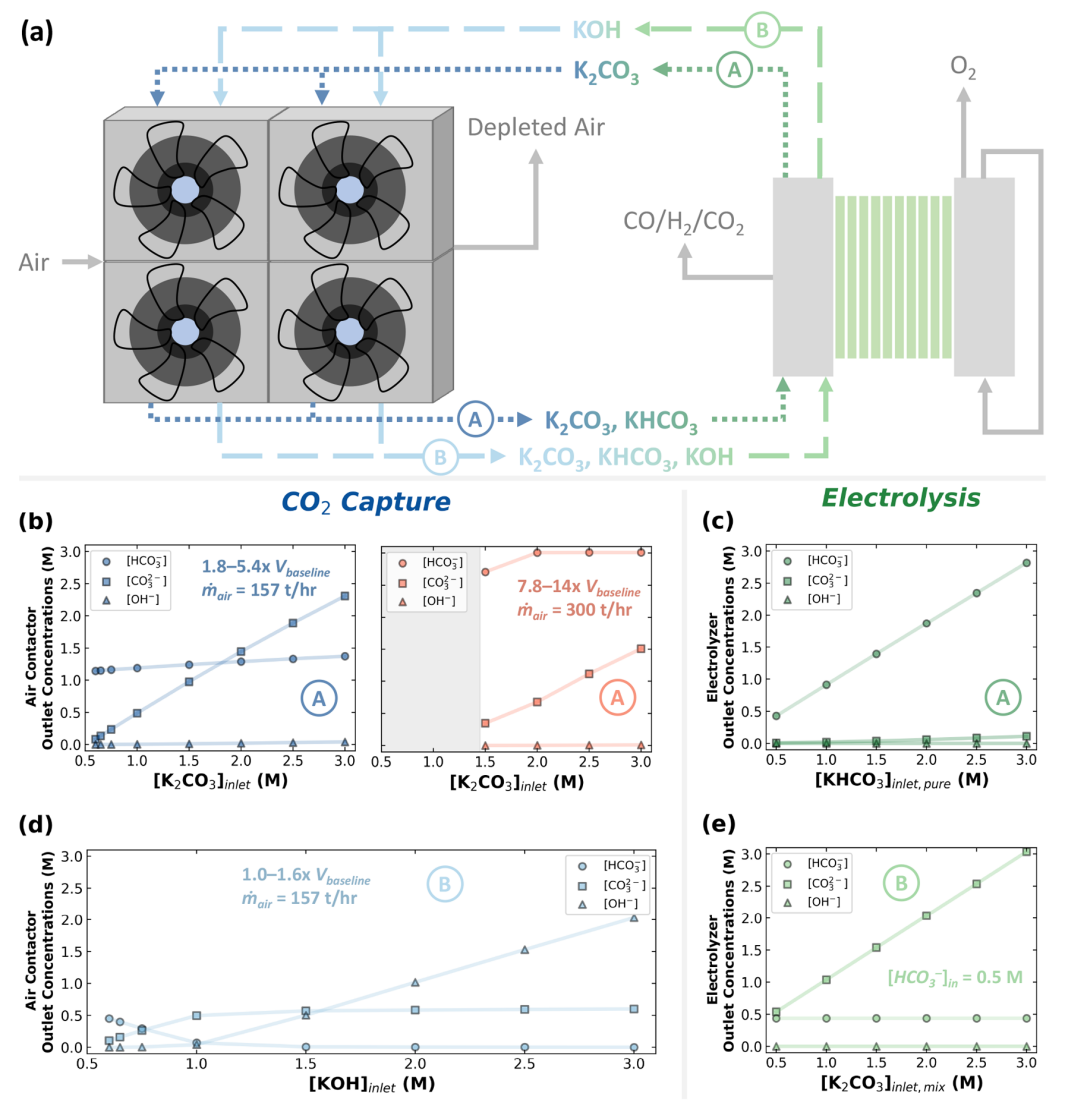
(a) Schematic of the literature-proposed integration route showing the air contactors on the left and the electrolyzer stacks on the right. Note the two possible pathways: (A) K_2_CO_3_-based and (B) KOH-based capture. The air contactor outlet concentrations of HCO_3_^–^, CO_3_^2–^, and OH^–^ as a function of the air contactor inlet concentration of (b) K_2_CO_3_ and (d) KOH are shown. The right-hand plot in (b) shows the outlet anionic species concentrations after increasing the air flow rate and contactor volume for the inlet K_2_CO_3_ concentrations of 2–3 M. The catholyte outlet concentrations of HCO_3_^–^, CO_3_^2–^, and OH^–^ as a function of the catholyte inlet concentration of (c) KHCO_3_ and (e) K_2_CO_3_/KHCO_3_ mixtures are also shown. For all air contactor calculations, except for (b)-right, we assume a CO_2_ absorption rate of 646 t-CO_2_/yr at a CO_2_ capture fraction of approximately 78%. For (b)-right, the CO_2_ absorption rate is 1340–1454 5-CO_2_/yr and the CO_2_ capture fraction is 85–93%.

Indeed, the requirements for designing a circular capture-conversion process are uncertain for two reasons. First, for a (bi)carbonate electrolyzer to regenerate the capture solvent, much of the reactor will move away from optimal operating conditions (e.g., 3.00 M KHCO_3_ for CO production), resulting in poorer overall CO partial current densities and FE_CO_. Second, if the (bi)carbonate electrolyzer cannot fully regenerate the same alkaline solvent concentrations, then the size of the air contactor must be increased to capture the same amount of CO_2_, but with more sluggish kinetics due to reduced alkalinity. These trade-offs are critical to the design of potential integrated routes, but have yet to be addressed. Specifically, the major focus of integrated capture-and-conversion systems has been centered on the ability of a (bi)carbonate electrolyzer to form the desired products while ignoring its ability to regenerate the capture solvent concentrations and pH.

In this work, we directly address this knowledge gap by describing the practical trade-offs between the performance of air contactors and the performance of (bi)carbonate electrolyzers to identify the physical, economic, and practical challenges faced by the direct integration route, as shown in Figure 2a. Our results, drawn from mass-balance, microkinetic, and multiphysics modeling, underscore the inability of (bi)carbonate electrolyzers to regenerate the desired solvent concentrations and pH. We show that the CO_2_ capture fraction significantly decreases with time, demonstrating the effect of HCO_3_^–^ accumulation on the CO_2_ capture ability of the electrolyzer outlet stream. In addition, our contactor sizing calculations elucidate the necessary increase in contactor volume depending on the solvent choice and input concentration. Finally, we demonstrate that a practical capture-conversion system requires the addition of external pH adjustment steps, which could negatively influence the economics of the integrated pathway. Our high-level analysis can guide the field toward the most relevant research targets for integrating DAC with carbon-based electrolysis; thus contributing to meeting carbon neutrality targets as we approach the middle of the century.

## Mass Balances of the Air Contactor and (Bi)carbonate Electrolyzer

The direct integrated route of capture and conversion of atmospheric CO_2_ is shown in Figure 2a, where air contactors are envisioned to be integrated with the electrolyzer stacks. To realize this integration, two conditions must be satisfied. First, the air contactor liquid effluent needs to produce a (bi)carbonate mixture with a neutral or mildly alkaline pH before feeding into the electrolyzer. This condition has been shown to allow (bi)carbonate electrolyzers to achieve reasonably high FE_CO_ and CO partial current densities at relatively low cell voltages.^21−24^ Second, the electrolyzer cathodic outlet needs to regenerate the alkaline solvent at a high pH, as needed by the air contactor. This condition enables fast recapture of fresh CO_2_ from the atmosphere^5^ and low capital costs of the air contactor, as will be shown later in this work. It is important to note that the pH of a solution is related to the proton concentration in the same solution. Thus, performing a mass balance using the concentration of species is necessary to determine the pH of the liquid streams in our system.

To estimate the air contactor outlet composition with a changing solvent concentration, we use a verified DAC plant model from our previous work.^13^ To fairly compare the mass-balance and equipment sizing results, we fix the captured CO_2_ rate at 646 t-CO_2_/yr, comparable to the CO_2_ capture rate of a single air contactor unit as developed by Keith and colleagues.^5^ We assume constant flow rates of the air and liquid solvent inlets, unless otherwise noted. To capture the same amount of CO_2_ under these conditions, with different compositions and concentrations of the solvent, we vary the length of the air contactor, which is directly proportional to its volume. We further consider a specific case in which we vary the inlet air flow rate and the contactor length to produce approximately 3.00 M HCO_3_^–^ in the contactor effluent stream, which is the optimal HCO_3_^–^ concentration ([HCO_3_^–^]) demonstrated for liquid-based CO_2_ electrolysis to CO.^21,24^ In this case, the amount of absorbed CO_2_ is increased due to increasing the air mass flow rate from 157 to 300 t-air/h such that the [HCO_3_^–^] reaches the desired 3.00 M value.

Throughout this work, we consider two routes: (A) the integration of a K_2_CO_3_-based air contactor with an electrolyzer that is fed with KHCO_3_ and (B) the integration of a KOH-based contactor with an electrolyzer is fed with a mixture of K_2_CO_3_ and KHCO_3_. For simplicity, we assume no loss of potassium ions during the cyclic process. The first route captures CO_2_ using a K_2_CO_3_ solution, which forms KHCO_3_ (eq 1) or K^+^ and HCO_3_^–^ ions (eq S.23) in the aqueous phase (more detailed review of CO_2_ capture by K_2_CO_3_ can be found elsewhere^18,29^). The aqueous solution is then sent to a BPM electrolyzer to generate CO_2_*in situ* using 1 mol of H^+^ per mole of HCO_3_^–^ (eq 2). The *in situ* CO_2_ is finally reduced electrochemically to form CO and carbonates using HCO_3_^–^ as a proton source (eq 3), which is found at appreciable concentrations (≥0.5 M) and high current densities (≥100 mA/cm^2^) near the catalyst layer due to the neutralization of the alkaline reaction products (CO_3_^2–^/OH^–^) by the protons conducted through bipolar membrane or cation exchange membrane.^25,26^ More information can be found in section S.10 of the Supporting Information.
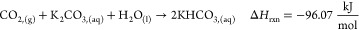
1
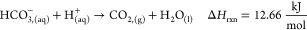
2
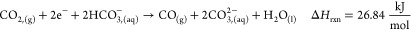
3The second route captures CO_2_ using KOH, forming K_2_CO_3_ as a main product (eq 4) or two K^+^ ions and one CO_3_^2–^ ion (eq S.24). The solution is then sent to a BPM electrolyzer to generate CO_2_*in situ*. However, 2 mol of H^+^ is now required per mole of CO_3_^2–^ to form CO_2_ (eq 5). Finally, the CO_2_ is electroreduced to CO and OH^–^ (eq 6) using H_2_O as a proton source. We note that HCO_3_^–^ ions could be intermediate products, which could possibly be used as proton sources for CO_2_ electrochemical reduction, as shown in eq 3. However, for simplicity, we do not consider HCO_3_^–^ as the proton source in K_2_CO_3_-based CO_2_ electrolysis. Details of the enthalpy of reaction calculations are provided in subsection S.8.2 of the Supporting Information.


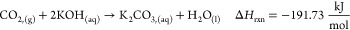
4
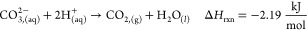
5
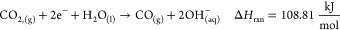
6

To begin our analysis, we examine the effect of the capture solvent choice and concentration on the resulting capture effluent composition, which is fed into the (bi)carbonate electrolyzer. Figure 2b (left) and Figure 2d show the outlet concentrations of the ionic species (i.e., [HCO_3_^–^], [CO_3_^2–^], and [OH^–^]) as a function of the contactor inlet [K_2_CO_3_] and [KOH], respectively. Again, we consider the cation to be K^+^ in all cases with no or negligible losses; thus, we focus on only the anionic species in the mass balances for simplicity. We notice that at low solvent concentrations of approximately 0.60–0.65 M, the captured effluent solution is mostly composed of HCO_3_^–^ ions with concentrations of 1.14 and 0.45 M for the K_2_CO_3_ (route A) and KOH (route B) cases, respectively. As the solvent inlet concentration increases from 0.60 to 1.00 M, [CO_3_^2–^] also increases in both cases from approximately 0.10 to 0.50 M. However, using a 1 M K_2_CO_3_ capture solvent mostly produces HCO_3_^–^ in the contactor effluent (1.19 M), whereas using the more alkaline 1 M KOH solvent mostly produces CO_3_^2–^ (0.50 M) with a small amount of HCO_3_^–^ (0.07 M).

Furthermore, Figure 2b (left) shows that the outlet [HCO_3_^–^] from the contactor does not significantly increase with higher [K_2_CO_3_] in the inlet stream. Indeed, a 3 M K_2_CO_3_ capture solution produces only 1.37 M HCO_3_^–^, which is below the 3.00 M target needed for electrolysis to produce CO at moderate to high FE_CO_ (≥60%).^24^ However, producing a [HCO_3_^–^] of 3.00 M in the captured solution requires almost doubling the air mass flow rate and significantly increasing the contactor length (∝ volume) by a factor of 7.75–14.32, as compared to the base 1 M KOH case (Figure 2b (right)). Although the contactor captures double the amount of CO_2_ in this specific case compared to the base cases and increases the capture fraction—i.e., the amount of captured CO_2_ over the amount of CO_2_ that enters the contactor—from 78% to 92%, its capital cost would be prohibitively high. Indeed, to produce bicarbonate at a concentration of 3.00 M, the air contactor capital cost would be 5–10 times that of the baseline case (1.00 M KOH, 646 t-CO_2_/yr, capture fraction ∼75%).

**Figure 3:**
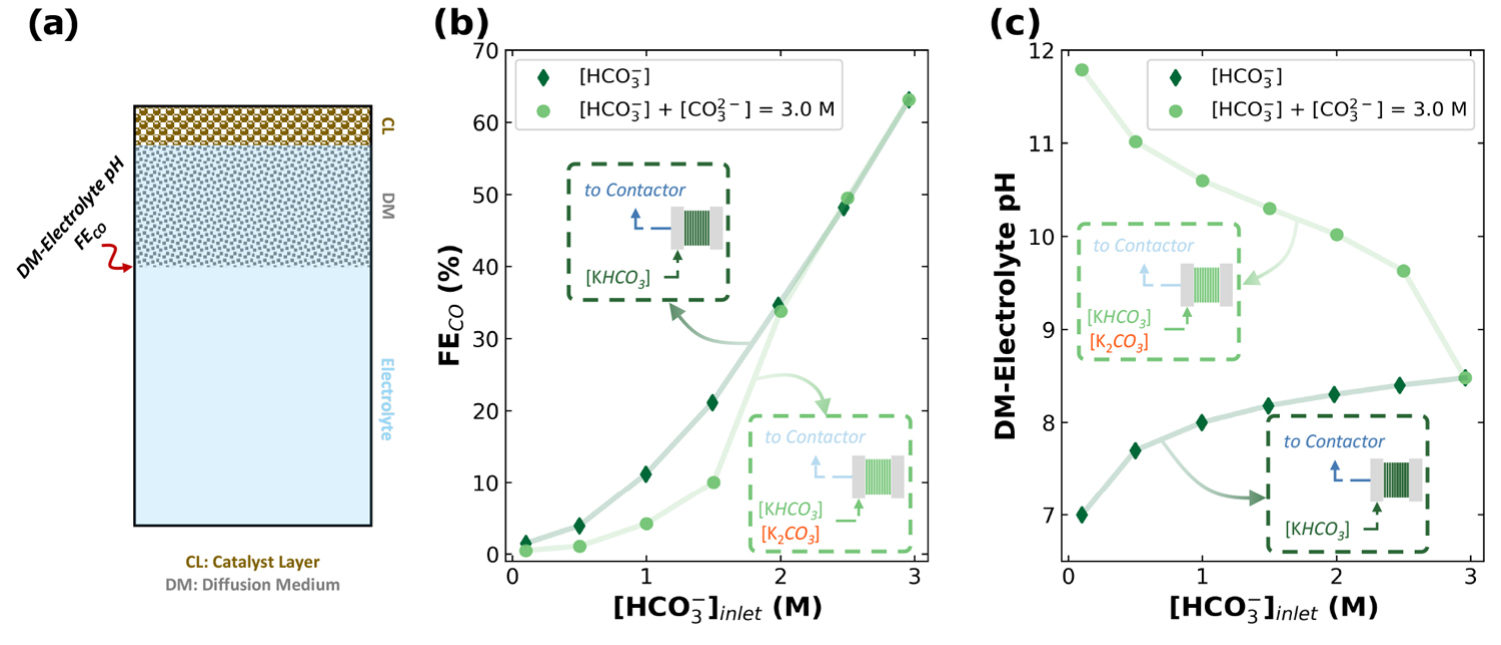
(a) Scheme of the 1D Multiphysics model. (b) Faradaic efficiency of CO and (c) DM–Electrolyte pH as a function of the inlet (bi)carbonate concentration to the electrolyzer. Note that the dark green diamonds represent the input of HCO_3_^–^ at different concentrations, whereas the light green circles represent the input of a mixture of HCO_3_^–^ and CO_3_^2–^ such that the total concentration is 3.00 M. For example, the circle at 2.00 M HCO_3_^–^ contains 1.00 M CO_3_^2–^ and 2.00 M HCO_3_^–^. In (b), we consider the pH at a 200 μm distance from the catalyst layer.

In addition, producing 3.00 M HCO_3_^–^ requires a contactor inlet [K_2_CO_3_] of ≥2.00 M and the copresence of 0.68–1.51 M CO_3_^2–^ due to the bicarbonate–carbonate equilibrium, which could significantly impact the electrolysis performance. To investigate this effect, we leveraged a detailed 1-D Multiphysics model^26^ to estimate the FE_CO_ at changing local concentrations of HCO_3_^–^ and CO_3_^2–^ ions (Figure 3b). It is worth noting that this model only considers a diffusion medium (DM) and a catalyst layer (CL), which might not allow it to capture some recent design developments, such as the addition of a catalyst diffusion adlayer.^27^ However, it can still be used to understand the general trade-offs between the HCO_3_^–^/CO_3_^2–^ concentrations and the electrolyzer performance metrics (e.g., FE_CO_).

Using our 1-D Multiphysics model, we observe that replacing HCO_3_^–^ with CO_3_^2–^ lowers the FE_CO_ significantly (Figure 3b), which is attributed to the reaction between CO_3_^2–^ and protons, and/or the increase in pH at the interface between the DM and bulk liquid electrolyte (DM–Electrolyte interface), as shown in Figure 3c. For instance, the replacement of 0.50 M HCO_3_^–^ with 0.50 M CO_3_^2–^ in a 3 M (bi)carbonate solution can lower the FE_CO_ from 63% to 49%—a 22% decrease in FE_CO_—and can increase the DM–Electrolyte pH from 8.48 to 9.63 (Figure 3b,c). Furthermore, as CO_3_^2–^ becomes the dominant ion in the solution (first four light green circles in Figure 3b,c), the local pH increases beyond 10.30, limiting the selective production of CO inside the electrolyzer. These findings are consistent with experimental results, as FE_CO_ is typically lower in carbonate solutions compared to that in bicarbonate (Table 1). Thus, compositional analyses of various air contactor capture effluents are needed to elucidate the suitability of integrating DAC with a (bi)carbonate electrolyzer. We note that although this type of analysis is uncommon in the DAC-electrolysis literature, it has been performed previously.^10^

To complete the mass balance of the integrated capture-conversion system, we now consider the overall electrolyzer mass balances. Figure 2c,e shows the outlet species concentration from the catholyte as a function of the inlet [KHCO_3_] and [K_2_CO_3_] to the electrolyzer, respectively. Note that Figure 2c assumes that the catholyte inlet is an almost pure KHCO_3_ solution, whereas Figure 2e assumes it is a mixture of 0.50 M KHCO_3_ and 0.50–3.00 M K_2_CO_3_. Another version of the figure where the input is pure K_2_CO_3_ is given in section S.11 of the Supporting Information. To perform these calculations, we utilized the same 1-D Multiphysics flow model, which was used to generate the results of Figure 3,^26^ and developed a microkinetic model to correlate the electrolyzer inlet KHCO_3_/K_2_CO_3_ concentrations with the outlet species concentrations. We integrate our mass balance calculations with our microkinetic model to roughly estimate the species concentrations in the flow channel. Our key assumptions are summarized in Table 2.

**Table 2:** Summary of Key Assumptions Used in the Microkinetic, Mass Balance, and 1D Multiphysics Models

parameter	value/note
operational current density	100 mA/cm^2^
CO_2_ conversion/utilization	100%
volumetric flow rate	100 mL/min
electrolyzer cell area	100 cm^2^
gas products	CO and H_2_
proton source (KHCO_3_ case)	HCO_3_^–^
proton source (KHCO_3_/K_2_CO_3_ case)	H_2_O
FE_CO_ (KHCO_3_ case)	3.9–63.2% (*from 1D Multiphysics model*)
FE_CO_ (KHCO_3_/K_2_CO_3_ case)	40%

We acknowledge that an electrolyzer cross-section area of 100 cm^2^ is larger than that used in all previous (bi)carbonate electrolysis experimental works in the literature (i.e., 1–4 cm^2^),^10,18,20−24,27^ but we assume future developments will enable the achievement of the same key performance metrics at this larger scale, as it is a necessary precursor for commercial viability. We also note that the FE_CO_ of the KHCO_3_/K_2_CO_3_ case is kept constant in our models at 40% because it is the highest experimentally achieved FE_CO_ for this system (Table 1). Lastly, it is worthwhile to define the CO_2_ conversion/utilization, which is simply the carbon efficiency, as the number of moles of carbon in the output CO over the number of moles of carbon in the *in situ* generated CO_2_ (eq S.26).

We find that the [OH^–^] and [CO_3_^2–^] in the electrolyzer outlet are too low to recapture CO_2_ from the atmosphere (Figure 2c). For all of the tested inlets [KHCO_3_] to the electrolyzer, we estimate the maximum [OH^–^] and [CO_3_^2–^] to be 0.07 × 10^–4^ and 0.11 M, respectively. Indeed, the outlet pH values of all cases in Figure 2c are mildly alkaline, ranging from 8.56 to 8.90. To put this into context, the ocean, which captures CO_2_ slowly based on its equilibrium with the atmosphere, has a pH value between 8.1 and 8.3.^30^ In addition, we find the outlet [HCO_3_^–^], balanced by [K^+^], to be almost the same as the inlet [KHCO_3_] when the electrolyzer is fed with an almost pure KHCO_3_ solution, signifying the very low HCO_3_^–^ conversion to CO_2_, which is consistent with the calculations by Lees et al.^21^ We reason that this observation is due to the HCO_3_^–^ acting as both an ion-conducting electrolyte and a key reactant, limiting the electrolyzer’s ability to fully consume it (Figure 2c). Consequently, the regenerated OH^–^ ions from the electrochemical CO_2_ reduction reaction (eq 6) per electrolyzer pass is negligible, favoring their consumption near the catalyst surface to maintain the chemical equilibrium between CO_2_, HCO_3_^–^, and CO_3_^2–^.

Similarly, we find that [OH^–^] in the carbonate electrolyzer outlet is too low to be recycled for further direct air capture (Figure 2e). However, since we are linearly increasing the inlet [K_2_CO_3_] while keeping the inlet [KHCO_3_] constant, we observe a linear increase in the outlet [CO_3_^2–^], resulting in a high pH range of the electrolyzer outlet, ranging from 10.40 to 11.15. While the high observed pH at the outlet may suggest the possibility of fresh CO_2_ being recaptured from air, it is important to highlight that the pH values at the inlet of the electrolyzer are in a similar range of 10.31–11.09, emphasizing the marginal increase in pH inside the electrolyzer.

Additionally, the presence of HCO_3_^–^ ions, as shown in Figure 2e, in the electrolyzer outlet stream could slow the sequential capture process, requiring even larger air contactors than what were predicted in Figure 2b (left). In fact, the use of a K_2_CO_3_-rich capture agent requires extremely large air contactors, which will be shown later, due to the sluggish kinetics of CO_2_ capture by K_2_CO_3_ as compared to KOH. These trade-offs must be carefully considered during the design phase to avoid potential operational challenges of the integrated process.

## Effect of HCO_3_^–^ Accumulation on the Recapture of Fresh CO_2_

To understand the behavior of an integrated capture-and-conversion unit as a whole, we used our mass-balance and microkinetic models to estimate the electrolyzer outlet pH as a function of simulation iteration, where a single iteration refers to the single passage of the liquid solvent through both the air contactor and the electrolyzer. For simplicity, we assume a fixed FE_CO_ of 40% in the electrolyzer and a steady-state operation in both the contactor and electrolyzer. Initially, we flow 1.00 M K_2_CO_3_ solution in the air contactor and allow it to change based on the ability of the electrolyzer to regenerate the capture solvent.

**Figure 4:**
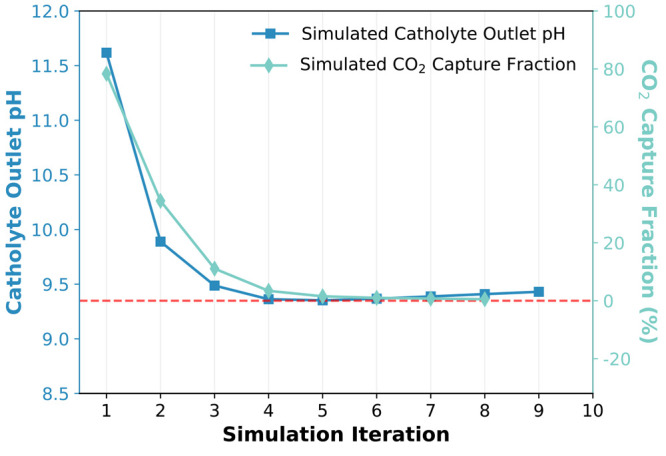
Simulated electrolyzer catholyte outlet pH (left *y*-axis; blue) and CO_2_ capture fraction (right *y*-axis; turquoise) as functions of iteration. Note that the red dashed horizontal line highlights the 0% CO_2_ capture fraction and a minimum catholyte outlet pH of about 9.35.

Our calculations show a decreasing catholyte outlet pH from 11.62 to 9.35 with simulation iteration (Figure 4), consistent with previous experimental observations of the same system.^18^ More importantly, we find that the CO_2_ capture fraction decreases from a maximum of 78.34% to a minimum of 0.52% with the simulation iteration (Figure 4). Indeed, after the fifth iteration, the CO_2_ capture fraction is already less than 1%. It is worth noting that previous experiments obtained a similar result, showing a significant decrease in CO_2_ capture rate at a pH of 9.1.^18^ The poor CO_2_ capture behavior can be explained by the buildup of HCO_3_^–^ ions in the catholyte outlet stream and the presence of the bicarbonate–carbonate equilibrium inside the electrolyzer, which are all accounted for in our integrated models. More precisely, as the pH is reduced to a mildly alkaline value of 9.35, the catholyte outlet becomes unsuitable for further CO_2_ capture due to outgassing of CO_2_ in the air contactor.^31,32^ Further details are given in section S.9 of the Supporting Information.

## Economic Implications of the Literature-Proposed Integrated Route

So far, we have demonstrated the incompatibility of the *direct* integration of air contactors with (bi)carbonate electrolyzers while producing CO selectively and recapturing CO_2_ continuously from the atmosphere. In this section, we aim to understand the effects of the presented mass balances on the capital cost of the system, regardless of the capture and conversion performance. We use the same methodology as in Mass Balances of the Air Contactor and (Bi)carbonate Electrolyzer, and we choose the basis for the cost comparison to be Carbon Engineering’s air contactor, as presented by Keith et al., which was optimized to capture 646 t-CO_2_/yr using a 1 M KOH solvent.^5^

**Figure 5:**
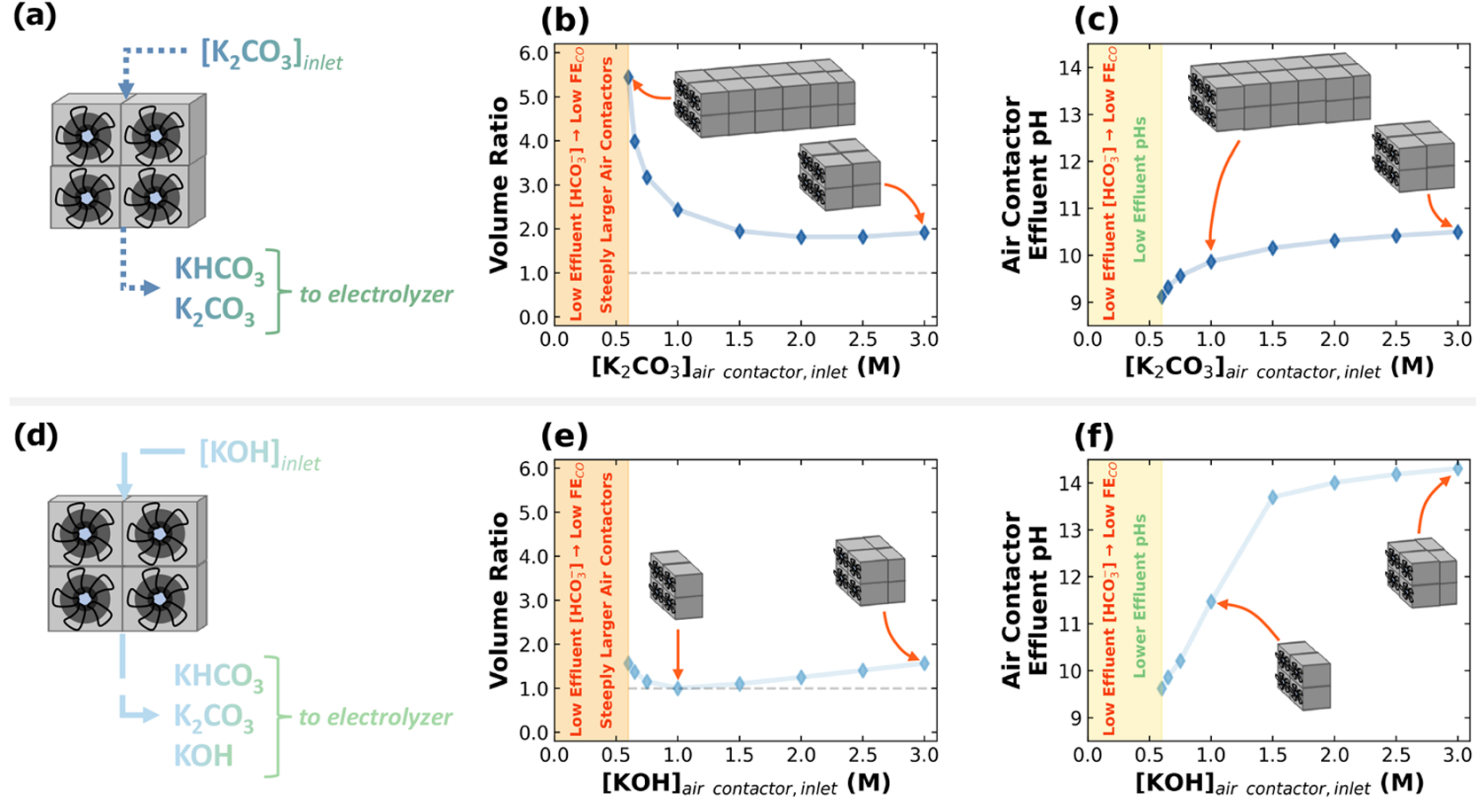
Schemes of (a) K_2_CO_3_-fed and (d) KOH-fed air contactors. Air contactor volume ratios of (b) K_2_CO_3_-fed and (e) KOH-fed air contactors as functions of the solvent concentration. Air contactor effluent pH as a function of inlet air contactor concentrations of inlet (c) K_2_CO_3_ and (f) KOH solvents. Note that the drawings of the air contactors indicate the size with respect to the 1 M KOH contactor. For instance, the first point in (b) from the left (0.60 M K_2_CO_3_ solvent) corresponds to 5.5 times the size of a contactor operating with 1 M KOH solvent, considering the same capture rate of 646 t-CO_2_ per year. In all of these cases, the capture fraction was approximately 78%.

Figure 5b,e shows the volume ratio as a function of the contactor’s inlet [K_2_CO_3_] and [KOH], respectively. Note that we fix the capture rate and define the volume ratio according to eq 7, where  is the molarity of the solvent. Figure 5c,f presents the contactor effluent pH as a function of the inlet contactor solvent concentrations.
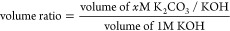
7

We find that using 0.65–3.00 M K_2_CO_3_ solvents results in contactor volumes that are 1.8–5.4 times larger than those of a typical Carbon Engineering unit (Figure 5b). On the other hand, we find that using 0.65–3.00 M KOH capture solvents requires only up to 1.6 times the baseline contactor volume to capture the same amount of CO_2_ at the same liquid-to-gas volumetric/mass ratio (Figure 5e). The low contactor volume ratio of this system is directly influenced by the faster CO_2_ capture kinetics of the KOH-based (Figure 5d) system as compared to that of the K_2_CO_3_-based system (Figure 5a). This result suggests that significantly fewer total capital expenditures of the contactor are needed for an integrated route that uses KOH as a capture agent as opposed to one that uses K_2_CO_3_ instead.

Notably, we find that K_2_CO_3_ solvent concentrations of less than 0.65 M require significantly high contactor volumes. This result demonstrates that a 3.00 M bicarbonate electrolyzer that produces any amount less than 0.65 M of K_2_CO_3_ is completely infeasible to integrate with CO_2_ capture, as it will demand significantly large air contactors to capture the same amount of CO_2_. Indeed, considering HCO_3_^–^ as the proton source for the electrochemical reduction of *in situ* CO_2_ to CO,^26^ 32.5% of HCO_3_^–^ will need to be converted to generate enough CO_3_^2–^ for possibly feasible integration. This conversion is far from state-of-the-art bicarbonate electrolysis devices used today, which convert less than 1% of the HCO_3_^–^ feed,^21^ likely due to HCO_3_^–^ acting as both a catholyte and a reactant.

In the case of using a 1 M K_2_CO_3_ solvent, which was recently tested experimentally,^18^ we find the required contactor volume to be 2.44 times that needed for a 1 M KOH solvent to capture 646 t-CO_2_/yr at a fixed air feed flow rate of 157 t/h. At these conditions and at a cell voltage of 3.3–3.5 V, we estimate the air contactor and (bi)carbonate electrolyzer capital costs to be approximately $_2023_582000 and $_2023_352000–373000, respectively. These numbers are equivalent to 2.14 times the baseline contactor capital cost and 3.40–3.60 times the capital cost of a typical low-temperature CO_2_ electrolyzer,^13,33^ respectively (see section S.7 in the Supporting Information). Note that the (bi)carbonate electrolyzer in this route produces CO at a low selectivity of 40%,^18^ which is likely due to the presence of both HCO_3_^–^ and CO_3_^2–^ species (see Figure 3). Lowering the amount of CO_3_^2–^ in the capture effluent (and thus, the catholyte inlet) requires a lower concentration of the K_2_CO_3_ solvent, allowing HCO_3_^–^ to be more dominant in the mixture (Figure 2b). However, a low-concentration K_2_CO_3_ solvent would require a larger air contactor to capture the same amount of CO_2_ per year (i.e., 646 t-CO_2_/yr). Indeed, we find that using a 0.75 M K_2_CO_3_ solvent increases the capital cost of the air contactor to $_2023_742000, approximately 2.73 times the capital cost of the baseline contactor. These results show that the feasible operation of the direct integrated system requires a significant capital cost increase in the air contactor, which could cause the overall economics of this pathway to be unfavorable.

Although special cases of the direct integrated route could enhance the economics of the capture-and-conversion system,^17,28^ one should still be aware of the technical challenges associated with the mass balances and stability of this direct integration. Our results demonstrate the important influence of the solvent choice on the contactor volume and, thus, the total capital cost of the integrated system. Therefore, future TEA studies should thoroughly evaluate the complete capture-and-conversion process instead of individual unit operations, as design choices in one unit may have significant up- or downstream economic consequences.

## Potential Solutions to the Integrated Capture-and-Conversion System

Overcoming the infeasibility of the direct integration of air contactors with (bi)carbonate electrolyzers requires careful consideration of mass-balance and operational limitations. Table 3 summarizes the operational parameters of air contactors and (bi)carbonate electrolyzers, which include the temperature, pressure, pH of the two connecting streams (i.e., catholyte inlet/outlet or contactor inlet/outlet), and electricity consumption. Based on literature values,^5,13,21,22,27^ the capture and conversion units are possibly compatible in terms of operational temperature and pressure. However, we find the pH values of the two connecting streams to be mostly incompatible. For instance, our results show that the pH range of the KOH-based air contactor inlet (i.e., 13.70–14.00) to not match well with the expected pH of the KHCO_3_/K_2_CO_3_-fed electrolyzer’s outlet (i.e., 10.40–11.15), necessitating the inclusion of pH treatment steps between the capture and conversion units. Additionally, it is worthwhile to note that the electricity consumption of the electrolyzer is more than 2 orders of magnitude higher than that of the air contactor. Although this difference does not present a compatibility issue, it signifies the electrolyzer’s dependence on electricity-related metrics (e.g., voltage, electricity price), which could impact the overall process economics and practical feasibility.

**Table 3:**
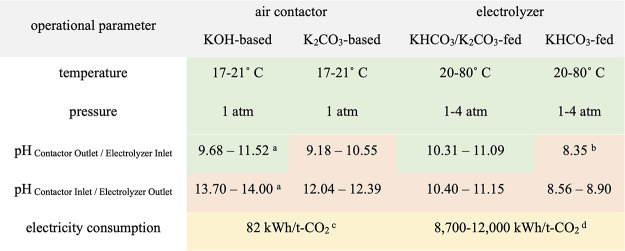
Summary of the Operational Parameters of the Air Contactor and (Bi)carbonate Electrolyzer^e^

a This range was calculated based on the KOH concentration range of 0.60–1.00 M to keep pH_maximum_ at 14.

b This pH was calculated using our microkinetic model.

c Taken from Keith et al.,^5^ assuming 70% fan efficiency and 82% pump efficiency.

d Estimated from our electrolyzer process model, considering *V*_minimum_ = 2.5 V and *V*_maximum_ = 3.5 V.

e Green and red cells highlight compatibility and incompatibility, respectively. Yellow cells show an important difference between the units.

**Figure 6:**
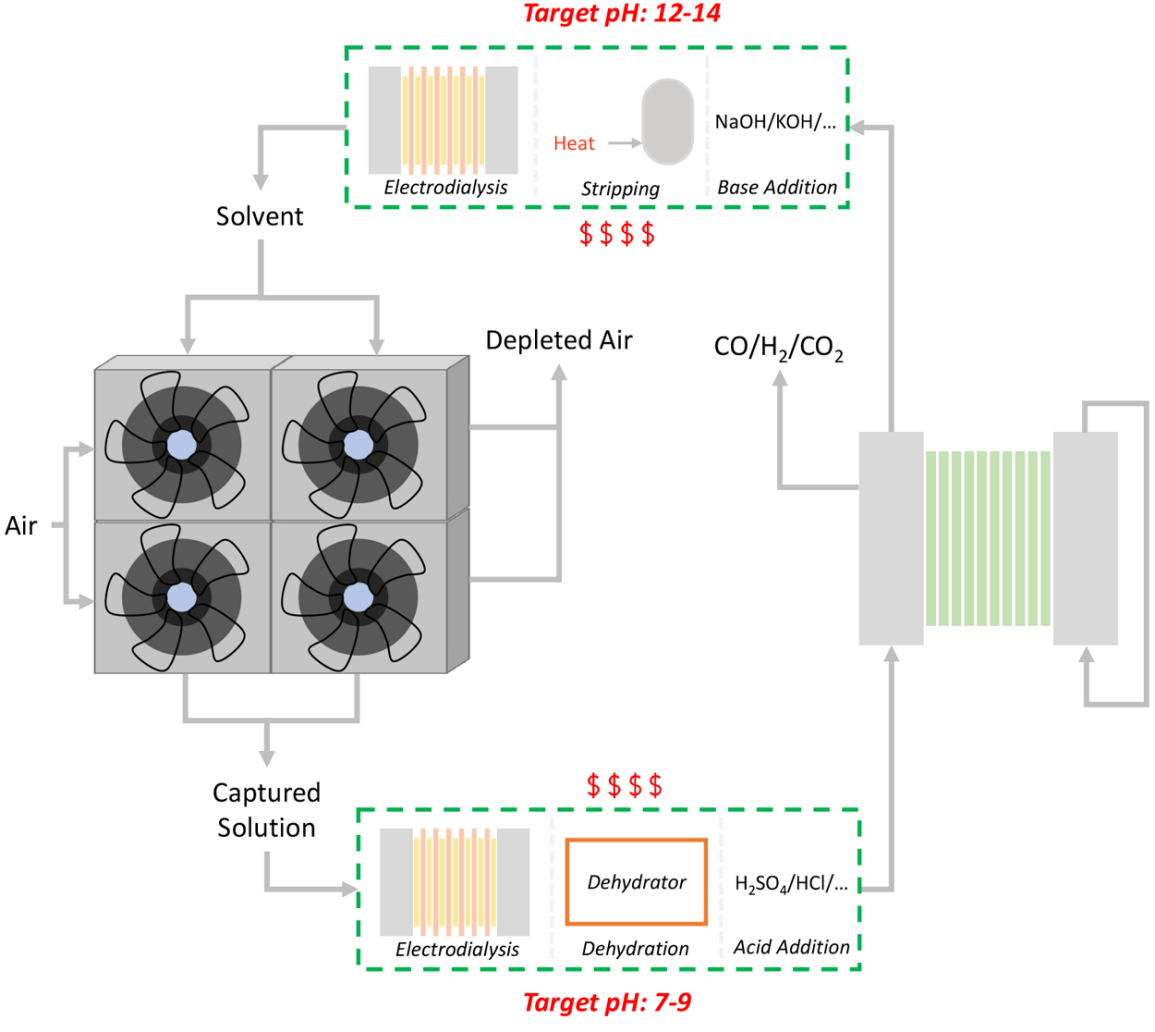
Schematic of the literature-proposed integration route, with some potential solutions shown inside the green dashed boxes. On the left side, we show the air contactors and on the right side are the (bi)carbonate electrolyzer stacks. At the top and bottom, we show potential solutions that could satisfy the different pH requirements of the capture and conversion processes, which include electrodialysis, evaporator, and an acidic stream on the bottom and electrodialysis, a stripping/heating step, and a basic stream on the top.

Therefore, we propose key modifications, as shown inside the green dashed boxes in Figure 6. First, increasing the [HCO_3_^–^] to 3.00 M in the contactor effluent while maintaining a mildly alkaline or neutral pH is needed to achieve high electrolyzer performances (Table 1). This step can be performed inside or outside the air contactor. We demonstrated that high capital costs will be required for producing a highly concentrated HCO_3_^–^ stream inside the contactor (Figure 2a; right). We also demonstrated that the copresence of CO_3_^2–^ might not be the best option for optimal electrolysis performance (Figure 3b). Thus, it is worthwhile to consider solutions that produce pure 3 M KHCO_3_*outside* of the contactor such as

- acidifying the contactor effluent stream using the acid stream from an electrodialysis unit
- acidifying the contactor effluent stream by feeding a continuously supplied acidic stream
- dehydrating the solution to increase the contactor outlet [HCO_3_^–^]

Second, the catholyte outlet needs to be able to recapture CO_2_ from the inlet gas stream. Earlier, we showed that the KHCO_3_-fed electrolyzer produces a low-pH stream with a low OH^–^ content (Figure 2c). Additionally, we showed that operating this integrated system with a 1 M K_2_CO_3_ capture solvent will likely accumulate bicarbonate, reducing the CO_2_ capture fraction (Figure 4). Therefore, potential solutions to these issues include

basifying the electrolysis outlet stream using the basic stream from an electrodialysis unit
basifying the electrolysis outlet stream by feeding a continuously supplied basic stream
heating the catholyte outlet to 80–100 °C, similar to the procedure done in the Benfield process,^34^ to degas CO_2_ from the (bi)carbonate mixture; thus increasing its pH

Moreover, improving the electrolyzer and contactor designs *simultaneously* could break the restrictions presented in this work. Particularly, designing contactors that maximize [HCO_3_^–^] in the effluent stream and designing electrolyzers that perform well independently of the inlet [HCO_3_^–^] could enable a practical integration of air contactors with (bi)carbonate electrolyzers. However, achieving both targets can be challenging, especially when considering the CO_2_–HCO_3_^–^–CO_3_^2–^ equilibrium.

Overall, the solutions that would benefit this integrated route require the acidification of the contactor effluent and the basification of the regenerated solvent. We note that adding an electrodialysis unit that supplies an acidic stream to the former and a basic stream to the latter might be sufficient, but it will add to both the capital and the operational costs. Indeed, all presented approaches would require additional capital and operational costs that might limit the economic feasibility of the integrated system.^35^ However, adding an electrodialysis unit might present an additional economic challenge when the system is integrated with renewables simply due to the high sensitivity of electrochemical processes to the price volatility of solar- and wind-based electricity. Therefore, further thorough TEA studies that consider the additional equipment needed, operational challenges, and variability of renewable electricity prices are necessary to improve our understanding of the economic feasibility of the presented (and similar) integrated capture-and-conversion routes. However, future TEA studies must be based on rigorous mass-balance models to ensure practical feasibility of the proposed process designs. Additionally, experimental DAC-electrolysis studies need to confirm the electrolysis performance with realistic capture effluents. Specifically, the continuous ability of the electrolyzer outlet solution to recapture CO_2_ from the atmosphere for tens of thousands of capture-and-conversion cycles is still missing from the literature, but critical for the potential impact of these technologies.

The urgency of achieving net-zero carbon emission goals due to the serious impacts on people around the world from the devastating effects of climate change necessitates the careful pursuit of research in rapidly developing carbon-neutral and carbon-free technologies. The direct integration of air contactors with (bi)carbonate electrolyzers has been proposed to be a cost- and energy-efficient pathway for air-to-products routes. Here, we demonstrate that this direct integration is practically infeasible without additional treatment steps. We presented mass balance calculations that illustrated the incompatibility of air contactors to be *directly* integrated with (bi)carbonate electrolyzers due to the different pH values needed for capture and conversion. In addition, we utilized our models to predict the CO_2_ capture fraction in the air contactor with time, which showed a significant decrease from 78% to less than 1%. Further, our analysis showed that producing a desired bicarbonate concentration that optimizes the performance of the electrolyzer requires significantly large air contactors, which would impact the premise of pursuing this integration. Indeed, the required air contactor volume for producing highly concentrated (2.70–3.00 M) HCO_3_^–^ was found to be 7–14 times larger than the state-of-the-art air contactor volume, which would cause the economics of this route to be unfavorable. Finally, we identified that acidifying the captured solution before feeding into the electrolyzer and basifying the catholyte outlet before feeding into the air contactor may solve the operational issues of this integrated route. One technology that could be promising but still needs further experimental, modeling, and technoeconomic investigations is bipolar membrane electrodialysis, which can supply acidic and basic streams from an inexpensive input of brine.

## Supporting Information Available

Air contactor process model, general approach to electrolyzer mass balance, multiphysics models, mass balance model, and microkinetic model, capital cost estimations (with an example calculation), change in enthalpy calculations, extra description of Figure 4, proton source discussion, supplementary figures, and carbon efficiency definition (PDF)
