# Supplementary material for: Closing the Loop: Unexamined Performance Trade-Offs of Integrating Direct Air Capture with (Bi)carbonate Electrolysis
Source: ACS Energy Lett. 2024 May 1;9(5):2472–83. doi: 10.1021/acsenergylett.4c00807 (PMC11091874; doi:10.1021/acsenergylett.4c00807)
Supplement: Supplementary file 1 — nz4c00807_si_001.pdf [file nz4c00807_si_001.pdf]

Supplementary Information: Closing the loop: Unexamined performance trade-offs of integrating direct air capture with (bi)carbonate electrolysis

Hussain M. Almajed[a,b], Recep Kas[c], Paige Brimley[a,b], Allison M. Crow[a,b,c], Ana Somoza-Tornos[d], Bri-Mathias Hodge[b,c,e,f], Thomas E. Burdyny[d], Wilson A. Smith[a,b,c,*]

[a] Department of Chemical and Biological Engineering, University of Colorado Boulder, Boulder, Colorado 80303, United States
[b] Renewable and Sustainable Energy Institute, University of Colorado Boulder, Boulder, Colorado 80303, United States
[c] National Renewable Energy Laboratory, Golden, CO, USA 80401
[d] Department of Chemical Engineering, Delft University of Technology, Van der Maasweg 9, 2629 HZ Delft, The Netherlands
[e] Department of Electrical, Computer and Energy Engineering, University of Colorado Boulder, Boulder, Colorado 80303, United States
[f] Department of Applied Mathematics, University of Colorado Boulder, Boulder, Colorado 80303, United States

*Corresponding Authors: Wilson.Smith@nrel.gov

Table of Contents

S.1. Air contactor process model ... 3

S.2. General approach to electrolyzer mass balance ... 5

S.3. Multiphysics model ... 6

S.4. Electrolyzer mass balance model ... 6

S.5. Microkinetic model ... 7

S.6. Capital cost estimations ... 8

S.7. Example calculation ... 10

S.7.1. CAPEX of the 1 M K_2CO_3 capture-and-conversion system ... 10

S.8. Change in enthalpy calculations ... 12

S.8.1. Dissociation of $KHCO_3$ and K_2CO_3 ... 12

S.8.2. Enthalpies of reactions ... 13

S.9. Extra details on Figure 4 ... 13

S.10. Justification of using HCO_3^- as a proton source for CO_2 electrolysis ... 15

S.11. Supplementary Figures ... 17

S.12. Carbon efficiency ... 18

References ... 19

S.1. Air contactor process model

We developed an air contactor process model in Aspen Plus in one of our previous works[1] and have modified it for the purposes of this study. In general, the model is based on Keith et al.'s[2] work on CO_2 capture from air using a liquid KOH/K_2CO_3 capture solvent. Our model leverages the Electrolyte Non-Random Two-Liquid (ELECNRTL) thermodynamic model in Aspen to account for like-ion repulsion and electroneutrality.[3] Following Keith et al.'s method,[2] we added the ASPENPCD, AQUEOUS, INORGANIC, PURE26, and SOLIDS databanks to the ELECNRTL default databanks.

We built the air contactor using a RADFRAC unit at 25 ˚ C and 1 atm with a constant solvent flow rate of 3,418 kg per hour and a constant air flow rate of 157 t-air per hour. At these conditions, we are fixing the amount of CO_2 that enters the air contactor per unit time. One of the key elements in this study was the constant CO_2 capture rate of 646 t-CO_2 per year, which allows us to perform a fair comparison of the KOH-based and K_2CO_3-based systems. In addition to the flow rate information, we fix the diameter of the contactor unit to 5 m and vary its length from 7 m to 110 m, depending on the concentration of the capture solvent. Table S.1. summarizes the design specifications of the contactor model. Further details about this model can be found in our previous work.[1]

Table S.1. Modeling specifications.

RADFRAC Specifications	
Number of Stages	16
Calculation Type	Rate-Based
Condenser?	No
Reboiler?	No
Valid Phases	Vapor-Liquid
Convergence Type	Standard

Pressure Drop	127.515 Pa
Liquid and Gas Feed Temperature	21 ˚C
Operating Liquid and Gas Feed Pressure	1 atm
Liquid Feed Stage	16, On-Stage
Air Feed Stage	1, On-Stage
Stage 1 Pressure	1 atm
**Column Specifications**	
Starting Stage	1
Ending Stage	16
Mode	Rating
Internal Type	Packed
Packing Type	MELLAPAK
Vendor	Sulzer
Material	Standard
Dimensions	250X
Section Packed Height	7 m
Diameter	5.642 m
**Design Specification**	
Mass Recovery Ratio	0.255
**Vary Parameter**	
Air Feed Rate	21,000 – 25,000 kmol/hr

Reaction Specifications	
Reactions Included	Equations (S.1) – (S.4)
Starting Stage	1
Ending Stage	16
Residence Time	0.001 sec

S.2. General approach to electrolyzer mass balance

The general approach that we follow to perform a full mass balance around the electrolyzer starts with estimating the Faradaic efficiency of CO (FE_{CO}) using a 1D multiphysics model, followed by a general mass balance of electrolysis, and ending with an estimation of the concentrations and pH values of relevant ionic and non-ionic species (*i.e.*, CO_2, H_2CO_3, HCO_3^-, CO_3^{2-}, OH^-, and H^+) using a microkinetic model. Figure S.1. summarizes this approach.

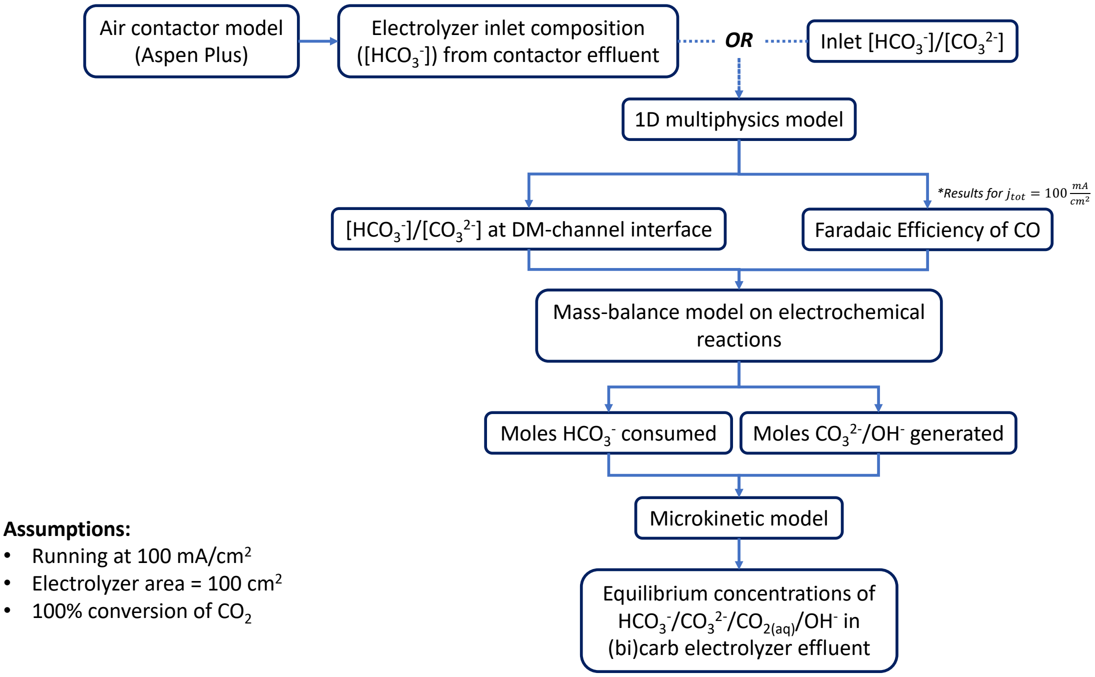

Figure S.1. General approach to perform electrolyzer mass balance.

S.3. Multiphysics model

Our 1D multiphysics model was built in COMSOL with the methodology presented in one of our previous works.[4] Generally, the model considers a diffusion medium (DM) and a catalyst layer (CL) that are flooded with a potassium bicarbonate at different concentrations. It predicts the concentration of ionic species as well as the pH of the solution near the DM-CL region, allowing for estimation of the Faradaic efficiency of CO. For a full set of reactions, rate constants, and rate equations, we refer the reader to the original work.[4]

S.4. Electrolyzer mass balance model

To perform a mass balance around the electrolyzer, we use the assumptions stated in Table S.2. and eq S.1 to calculate the total current of the electrolyzer. We then use this result to calculate the species mass flow rates and concentrations.

(S.1) $$i_{tot} = j_{tot} \bullet A_{electrolyzer} \bullet n_{cells} \bullet n_{stacks}$$

Table S.2. Rate constants of included reactions in the microkinetic model.

Metric	Value	Unit
$\dot{V}$	0.1	L/min
j_{tot}	100	mA/cm^2
$A_{electrolyzer}$	100	cm^2
Number of cells	100	cell
Number of stacks	138	stack

S.5. Microkinetic model

Our microkinetic model was built in Python, in which we use eq S.2–S.7 to generate species rate equations, eq S.8–S.13. We then utilize the backward differentiation formula (BDF) method to solve the set of ordinary differential equations, eq S.14–S.19, using the *solve_ivp* solver to estimate the steady-state concentrations of relevant ionic and neutral species (*i.e.*, H^+, OH^-, HCO_3^-, CO_3^{2-}, CO_2, and H_2CO_3). Table S.3. summarizes the rate constants used to build the microkinetic model.

(S.2) $$H_2O \leftrightarrow H^+ + OH^-$$

(S.3) $$CO_2 + OH^- \leftrightarrow HCO_3^-$$

(S.4) $$HCO_3^- + OH^- \leftrightarrow CO_3^{2-} + H_2O$$

(S.5) $$H_2CO_3 \leftrightarrow H_2O + CO_2$$

(S.6) $$H_2CO_3 + OH^- \leftrightarrow HCO_3^- + H_2O$$

(S.7) $$HCO_3^- + H^+ \leftrightarrow H_2CO_3$$

Table S.3. Rate constants of included reactions in the microkinetic model.

Rate constant	Value	Unit	Source
k_{1f}	2.29×10^{-1}	mol m^{-3} s^{-1}	[4]
k_{1b}	2.30×10^{7}	m^3 s^{-1} mol^{-1}	[4]
k_{2f}	2.23×10^{0}	m^3 s^{-1} mol^{-1}	[4]
k_{2b}	5.35×10^{-5}	s^{-1}	[4]
k_{3f}	6.00×10^{6}	m^3 s^{-1} mol^{-1}	[4]
k_{3b}	1.22×10^{6}	s^{-1}	[5]
k_{4f}	2.86×10^{1}	s^{-1}	[4]
k_{4b}	7.00×10^{-2}	s^{-1}	[4]
k_{5f}	1.00×10^{7}	m^3 s^{-1} mol^{-1}	[4]
k_{5b}	5.90×10^{-1}	s^{-1}	[4]
k_{6f}	6.50×10^{7}	m^3 s^{-1} mol^{-1}	[4]
k_{6b}	1.10×10^{7}	s^{-1}	[4]

(S.8) $$r_1 = k_{1f} - k_{1b}[H^+][OH^-]$$

(S.9) $$r_2 = k_{2f}[CO_2][OH^-] - k_{2b}[HCO_3^-]$$

(S.10) $$r_3 = k_{3f}[HCO_3^-][OH^-] - k_{3b}[CO_3^{2-}]$$

(S.11) $$r_4 = k_{4f}[H_2CO_3] - k_{4b}[CO_2]$$

(S.12) $$r_5 = k_{5f}[H_2CO_3][OH^-] - k_{5b}[HCO_3^-]$$

(S.13) $$r_6 = k_{6f}[HCO_3^-][H^+] - k_{6f}[H_2CO_3]$$

(S.14) $$\frac{d[H^+]}{dt} = r_1 - r_8$$

(S.15) $$\frac{d[OH^-]}{dt} = r_1 - r_2 - r_3 - r_5$$

(S.16) $$\frac{d[CO_2]}{dt} = -r_2 + r_4$$

(S.17) $$\frac{d[HCO_3^-]}{dt} = r_2 - r_3 + r_5 - r_6$$

(S.18) $$\frac{d[CO_3^{2-}]}{dt} = r_3$$

(S.19) $$\frac{d[H_2CO_3]}{dt} = -r_4 - r_5 + r_6$$

S.6. Capital cost estimations

In this work, we estimated the capital costs of the air contactor using the methodology presented by Towler and Sinnot.[6] We used eq S.20 and S.21 to estimate the contactor capital cost, adjusted to 2023 prices using the June 2023 chemical engineering plant cost index (CEPCI) of 803.3.[7] $f_{installation}$ is the installation factor of the equipment, summarized in Table S.4. C_{ref} is the reference price of the equipment (Table S.4). S_{ref} and S_{new} are the sizes of the reference and new equipment, which can be a volumetric rate or a volume in our case. n is the scaling factor, which is summarized in Table S.5 for different equipment. $C_{instrumentation}$ is the instrumentation cost if it was not accounted for in the installation factor (*e.g.*, for fans), also included in Table S.4. $C_{equipment}$ and $C_{equipment,2023}$ are the total cost of the equipment (*e.g.*, fans, pumps, etc.) in the referenced year and the current year, respectively.

(S.20) $$C_{equipment} = f_{installation} C_{ref} \left(\frac{S_{new}}{S_{ref}}\right)^n + C_{instrumentation}$$

(S.21) $$C_{equipment,2023} = C_{equipment} \left(\frac{CEPCI_{2023}}{CEPCI_{ref}}\right)$$

In addition to the contactor capital cost, we also estimated the electrolyzer capital cost by performing a mass balance around the bicarbonate- and carbonate-fed electrolyzers. We considered a basis of 646 t-CO_2 per year, which is the assumed capture rate of our air contactor. Apart from the electrochemical CO_2 reduction reaction, our electrolyzer process model accounts for the hydrogen evolution reaction (HER) and (bi)carbonate conversion to CO_2. The key result from our electrolyzer model is the electrolyzer power capacity ($P_{electrolyzer}$; in kW), which we use along with an assumed electrolyzer price ($C_{electrolyzer,ref}$) of $233.61 per kW to estimate the capital cost of the electrolyzer, (eq S.22). We note that the assumed electrolyzer price is consistent with the National Renewable Energy Laboratory's (NREL) H_2A production cost model for water electrolyzers in 2050.[8]

(S.22) $$C_{electrolyzer,2023} = P_{electrolyzer} C_{electrolyzer,ref} \left(\frac{CEPCI_{2023}}{CEPCI_{old}}\right)$$

It is worth noting that the capital costs estimated here do not represent a full economic analysis, but rather a portion of it, focusing on the capital cost of simply the contactor and electrolyzer. We also note that the capital cost is not annualized here, meaning it represents the total investment regardless of the discount rate or lifetime of the system. Again, these calculations are made to clarify an economic point, and are not meant to cover the full economics of the process. We refer the reader to Section 3 of the main text.

Table S.4. Reference costs and sizes, installation factors and instrumentation costs of equipment.

Equipment	Reference Cost	Reference Size	Installation Factor	Instrumentation Cost	Source
Centrifugal fans	$15,850	10 m^3/s	1.4	$7,000	[9]
PVC packing	$250	1 m^3	3.2	*Included in installation factor*	[10]
Pump	$28,102	137 L/s	4	*Included in installation factor*	[6]
H_2O/CO_2 electrolyzer	$233.61	1 kW	1.2	*Included in installation factor*	[8]

Table S.5. Scaling factors used of equipment.

Equipment	Scaling Factor
Centrifugal fans	0.78
PVC packing	1
Pump	1
H_2O/CO_2 electrolyzer	1

S.7. Example calculation

S.7.1. CAPEX of the 1 M K_2CO_3 capture-and-conversion system

The referenced cost of centrifugal fans, PVC packing, and pump are $15,850, $250, and $28,102, respectively. Their corresponding sizes are 10 m^3 per second, 1 m^3, and 137 L per second, respectively. A single contactor that captures 646 t-CO_2 per year with a 1.00 M K_2CO_3 solvent requires a fan with an air flow rate of about 37.13 m^3 per second, a PVC packing volume of 175 m^3 times 2.44 (i.e., *to account for volume difference*), and a pumped liquid

solvent of about 0.86 L per second. Using this information and eq S.19 and S.20, we get the following:

Centrifugal Fans:

$$C_{fans} = (1.4)(\$15{,}850)\left(\frac{37.13\,\frac{m^3}{s}}{10\,\frac{m^3}{s}}\right)^{0.78} + \$7{,}000 = \$68{,}736.68$$

- Note that we include instrumentation costs, following the source's method, because it is not accounted for in the installation factor

PVC Packing:

$$C_{packing} = (3.2)(\$250)\left(\frac{(2.44)(175\,m^3)}{1\,m^3}\right)^{1} = \$341{,}600$$

Pump:

$$C_{pump} = (4)(\$28{,}102)\left(\frac{0.86\,\frac{L}{s}}{137\,\frac{L}{s}}\right)^{1} = \$702.57$$

Extrapolating these costs to June 2023 costs would give:

Centrifugal Fans:

$$C_{fans,2023} = (\$68{,}736.68)\left(\frac{803.3}{1{,}000}\right) = \$55{,}216.18$$

- Note that the CEPCI of fans is 1,000, as given in Woods.[9]

PVC Packing:

$$C_{packing,2023} = (\$341{,}600)\left(\frac{803.3}{521.9}\right) = \$525{,}785.17$$

Pump:

$$C_{pump,2023} = (\$702.57)\left(\frac{803.3}{532.9}\right) = \$1{,}059.06$$

The total capital cost would then be:

$$C_{contactor,2023} = \$55{,}216.18 + \$525{,}785.17 + \$1{,}059.06 = \$582{,}060.41$$

For the bicarbonate-fed and carbonate-fed electrolyzers, we found the power capacities to be 898 and 847 kW, respectively. Thus, we can estimate the 2023 electrolyzers' capital costs as follows:

$$C_{electrolyzer,HCO_3^-} = (1.2)\left(\frac{\$233.61}{kW}\right)(898\ kW)\left(\frac{803.3}{541.7}\right) = \$373{,}457.59$$

$$C_{electrolyzer,CO_3^{2-}} = (1.2)\left(\frac{\$233.61}{kW}\right)(847\ kW)\left(\frac{803.3}{541.7}\right) = \$352{,}132.31$$

Following the same calculation method, we estimate the capital cost of the baseline contactor to be \$271,760.96, which uses 1 M KOH to capture 646 t-CO_2 per year. Additionally, for a gaseous CO_2 electrolyzer that utilizes 646 t-CO_2 per year, we calculate the power capacity to be 0.25, giving a capital cost of about \$103,675.66. Using these numbers, we can find the capital cost ratios to be 2.14 and 3.40-3.60, as mentioned in the main text.

S.8. Change in enthalpy calculations

S.8.1. Dissociation of $KHCO_3$ and K_2CO_3

The dissociation of $KHCO_3$ and K_2CO_3 simply happens when they are dissolved in water. The following equations (eq S.23 and S.24) present the balanced reactions:

(S.23) $$KHCO_{3,(aq)} \rightarrow K^+_{(aq)} + HCO^-_{3,(aq)} \qquad \Delta H_{rxn} = 20.90\,\frac{kJ}{mol}$$

(S.24) $$K_2CO_{3,(aq)} \rightarrow 2K^+_{(aq)} + CO^{2-}_{3,(aq)} \qquad \Delta H_{rxn} = -48.09\,\frac{kJ}{mol}$$

S.8.2. Enthalpies of reactions

To estimate the enthalpies of reactions, we used the enthalpies of formation (ΔH_f) that are summarized in Table S.6 and applied Hess' Law (eq S.25):

(S.25) $$\Delta H_{rxn} = \sum_{i,products} \nu_i \Delta H_{f,i} - \sum_{j,reactants} \nu_j \Delta H_{f,j}$$

Where ν is the stoichiometric coefficient of the respective species.

Table S.6. Enthalpy of formation of all relevant species.

Species	ΔH_f (kJ/mol)
$HCO^-_{3,(aq)}$	-691.99
$CO^-_{3,(aq)}$	-677.14
$H^+_{(aq)}$	0
$CO_{2,(g)}$	-393.51
$CO_{(g)}$	-110.54
$OH^-_{(aq)}$	-229.99
$H_2O_{(l)}$	-285.82
$KHCO_{3,(aq)}$	-963.20
$K_2CO_{3,(aq)}$	-1130.61

S.9. Extra details on Figure 4

We demonstrated in section 3 of the manuscript that the CO_2 capture fraction decreases with simulation iteration (or with time). Here, we provide a step-by-step explanation of our understanding of what is happening in each of these cycles:

1. Let's consider that the 1st simulation iteration starts with 1 M K_2CO_3 as an input to the contactor.

2. The 1 M K_2CO_3 will capture some amount of the CO_2 feed (say 78% of input CO_2; Figure 4 of the main text) to produce $KHCO_3$. The outlet will then contain some K_2CO_3 and $KHCO_3$, which will be directly sent to the electrolyzer.
3. The electrolyzer will get protons from the BPM and generate CO_2 *in-situ*, which we assume to be fully converted to CO (with a selectivity of 40%; *good assumption for carbonate electrolysis*). At this point, the outlet catholyte will be composed of both $KHCO_3$ and K_2CO_3 because the HCO_3^--to-CO_2 conversion is less than 1% of the input (bi)carbonate solution, as shown experimentally.[11] In other words, the catholyte input to the electrolyzer will be **almost the same** as the catholyte output from the electrolyzer (we see that in Figure 2c and 2e in the main text). This solution will then be sent to the contactor for the 2nd simulation iteration.
4. At this point, the pH of the outlet catholyte is still high due to the presence of carbonate ions (pH ≥ 11.5).
5. In the 2nd iteration, the contactor will not be able to capture as much CO_2 as it did in the 1st iteration because of the presence of a lower amount of K_2CO_3 (and KOH) compared to that in the 1st iteration. Indeed, we find the capture fraction to decrease to about 34%, according to our Aspen simulations (Figure 4 in the main text). This means that the contactor will not produce the same amount of $KHCO_3$ as in the 1st iteration, but rather a smaller amount. This solution is now sent to the electrolyzer.
6. The electrolyzer will utilize the same number of protons as in the 1st iteration (*assuming the applied current is constant*), but some of this amount will convert more carbonate to bicarbonate (and some will convert bicarbonate to CO_2 for CO production). Thus, we're now <u>reducing</u> the amount of carbonate, <u>roughly maintaining</u> the amount of bicarbonate, and <u>increasing</u> the amount of CO_2. Due to these effects and to the carbonate-bicarbonate equilibrium, the catholyte outlet should now have a lower pH value (≈ 10.5). The solution is then sent to the contactor for the 3rd iteration.
7. In the 3rd iteration, the solvent captures even less CO_2 in the contactor because we now have even more $KHCO_3$ than K_2CO_3 compared to the 2nd iteration. So, this

reduces the CO_2 capture fraction to 12% (Figure 4). The liquid contactor outlet is sent again to the electrolyzer.

8. The electrolyzer does a similar behavior as in step 6, which also reduces the pH value more (10, then 9.5, etc.) and subsequent cycles follow the same trend.
9. Eventually, the pH value will reach a point where the system accumulated enough $KHCO_3$ such that it cannot capture any more CO_2 inside the contactor. According to the experimental[12] and our simulation results, that pH is around 9.1-9.4, considering the equilibrium between carbonate, bicarbonate, and CO_2. Note that this pH region is still slightly alkaline, but not alkaline enough for CO_2 capture. At this pH, the electrolyzer should perform better than in the 1st simulation iteration (due to the accumulation of $KHCO_3$), which was indeed observed experimentally.[12]

S.10. Justification of using HCO_3^- as a proton source for CO_2 electrolysis

The production of hydrogen or CO from various proton sources, such as H_3O^+, HCO_3^-, and H_2O cannot be distinguished on a thermodynamically relevant scale, namely RHE. Nonetheless, the pK_a and the structure of the proton source dramatically influences the rate of reaction. Early CO_2ER studies did not provide clear differentiation because the bicarbonate concentrations were usually low, and the CO_2ER had not been examined under mass transfer-controlled conditions. Recently, it has been experimentally shown that phosphate ($H_2PO_4^-$) serves as a much more effective proton donor compared to H_2O during HER on gold (Au) surfaces.[13] Similar conclusions have been experimentally demonstrated in various studies on copper-gold electrodes during the CO_2ER.[14–16] The CO_2ER progresses through H_2CO_3, H_3O^+, and HCO_3^- as proton donors until a mass transfer limitation is reached, either due to the direct consumption or indirect consumption of the buffer ions. This typically manifests as a shoulder or plateau in the voltammetry curve at lower potentials than the water reduction (note that this should not be confused with CO adsorption or CO formation on copper

electrodes). In a conventional electrochemical cell with 1 M $H_2PO_4^-$, the plateau can be observed at current density as high as 100 mA/cm^2.[17] Moreover, in bicarbonate electrolysis cells, bicarbonate can be present at even higher current densities (200-300 mA/cm^2) near the catalyst layer due to the neutralization of the alkaline reaction products by the protons conducted through bipolar membrane or cation exchange membrane (CO_3^{2-}/OH^-).[4,18] Table S.7. summarizes the takeaways presented in this paragraph.

Table S.7. Summary of takeaways from literature regarding the proton source for HER/CO_2ER.

**Source**	**Takeaway regarding proton donors for HER/CO_2ER**
Resasco et al. (2018)[14]	"We propose that these residual differences are associated with the ability of buffering anions to serve as a significant source of hydrogen in competition with water." [a]
Jackson et al. (2019)[13]	"...phosphate, unlike borate, can outcompete water as a proton donor for interfacial CPET."
Marcandalli et al. (2021)[16]	"In an electrolyte concentration of 0.1 M Na^+, H_2O reduction may be preponderant, while for a concentration of 0.5 M Na^+, HCO_3^- reduction is the dominant branch of HER."
Marcandalli et al. (2022)[15]	"Through microkinetic modelling, we exclude that HER in bicarbonate can be explained via the generation of a proton by solution acid-base reactions."
Yang et al. (2019)[17]	Plateau of cell voltage at 100 mA/cm^2
Kas et al. (2022)[4]	"...this model suggests that the concentration of the bicarbonate does not reach a limiting value, both as a proton donor and CO_2 source, in the catalytically active regions of the bicarbonate flow cell. Since significant losses in electrocatalytic selectivity toward CO occur well below 100 mA cm^{-2}..., the decrease in HCO_3^- concentrations

	may not be the primary reason for the decline in FE, as a function of current density.”
Lees et al. (2022)[18]	“As the current density increases, the HCO_3^- concentration in the CEL decreases…because HCO_3^- reacts with H+ from water dissociation to form CO_2.” However, the HCO_3^- concentration maintains a value of > 1 M at current densities greater than 100 mA/cm[2]

[a] Buffering anions include HCO_3^-.

S.11. Supplementary Figures

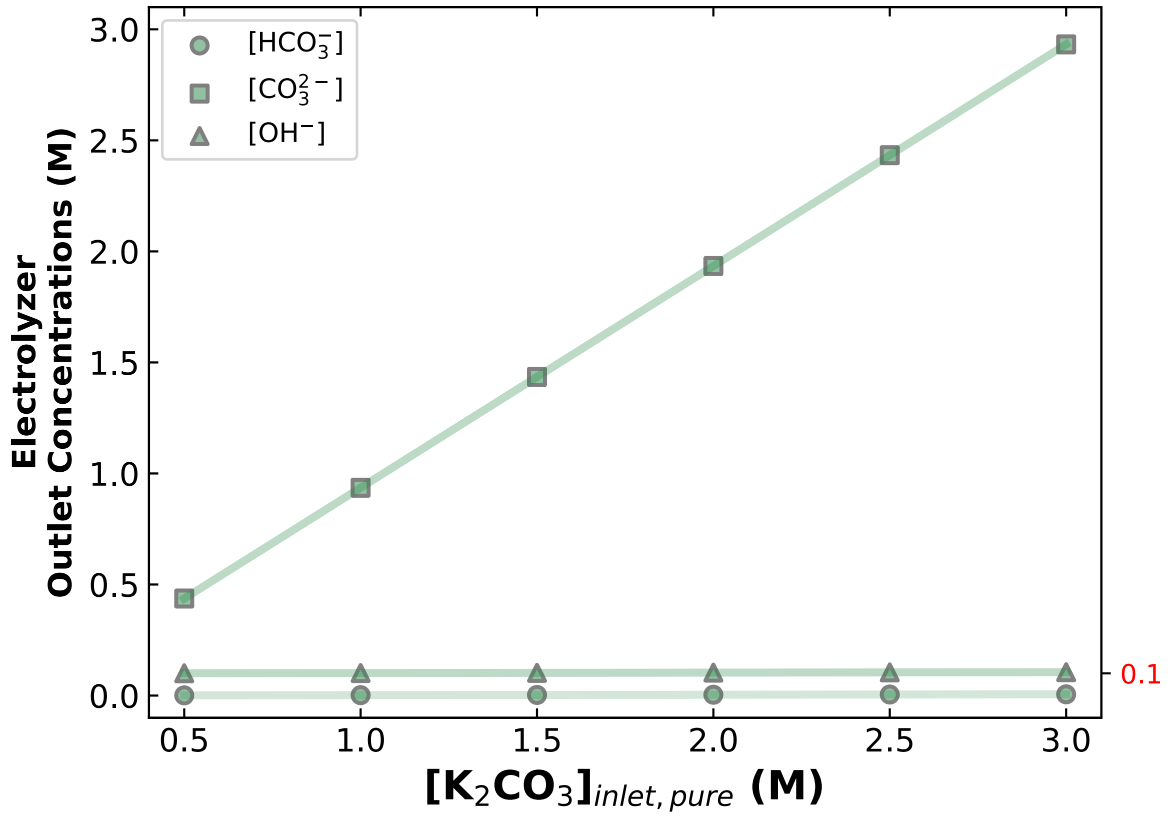

Figure S2. The catholyte outlet concentrations of HCO_3^-, CO_3^{2-}, and OH^- as a function of the catholyte inlet concentration of K_2CO_3, with 0 M $KHCO_3$.

S.12. Carbon efficiency

We define CO_2 conversion/utilization the same way we define carbon efficiency, using the following equation:

(S.26)

$$CE = \frac{n_{C,CO,out}}{n_{C,in-situ\,CO_2}} = \frac{n_{C,CO,out}}{n_{C,CO_2,out} + n_{C,CO,out}}$$

References

(1) Almajed, H. M.; Guerra, O. J.; Smith, W. A.; Hodge, B.-M.; Somoza-Tornos, A. Evaluating the Techno-Economic Potential of Defossilized Air-to-Syngas Pathways. *Energy Environ. Sci.* **2023**, *16* (12), 6127–6146. https://doi.org/10.1039/D3EE02589F.

(2) Keith, D. W.; Holmes, G.; St. Angelo, D.; Heidel, K. A Process for Capturing CO_2 from the Atmosphere. *Joule* **2018**, *2* (8), 1573–1594. https://doi.org/10.1016/j.joule.2018.05.006.

(3) Chen, C.-C.; Britt, H. I.; Boston, J. F.; Evans, L. B. Local Composition Model for Excess Gibbs Energy of Electrolyte Systems. Part I: Single Solvent, Single Completely Dissociated Electrolyte Systems. *AIChE J.* **1982**, *28* (4), 588–596. https://doi.org/10.1002/aic.690280410.

(4) Kas, R.; Yang, K.; Yewale, G. P.; Crow, A.; Burdyny, T.; Smith, W. A. Modeling the Local Environment within Porous Electrode during Electrochemical Reduction of Bicarbonate. *Ind. Eng. Chem. Res.* **2022**, *61* (29), 10461–10473. https://doi.org/10.1021/acs.iecr.2c00352.

(5) Cents, A. H. G.; Brilman, D. W. F.; Versteeg, G. F. CO_2 Absorption in Carbonate/Bicarbonate Solutions: The Danckwerts-Criterion Revisited. *Chem. Eng. Sci.* **2005**, *60* (21), 5830–5835. https://doi.org/10.1016/j.ces.2005.05.020.

(6) Towler, G.; Sinnott, R. Capital Cost Estimating. In *Chemical Engineering Design*; Elsevier, 2013; pp 307–354. https://doi.org/10.1016/B978-0-08-096659-5.00007-9.

(7) *Cost Indices – Towering Skills*. https://toweringskills.com/financial-analysis/cost-indices/ (accessed 2023-04-12).

(8) NREL. *H2A: Hydrogen Analysis Production Models*. https://www.nrel.gov/hydrogen/h2a-production-models.html (accessed 2023-02-20).

(9) Woods, D. R. Appendix D: Capital Cost Guidelines. In *Rules of Thumb in Engineering Practice*; John Wiley & Sons, Ltd, 2007; pp 376–436. https://doi.org/10.1002/9783527611119.app4.

(10) Holmes, G.; Keith, D. W. An Air–Liquid Contactor for Large-Scale Capture of CO_2 from Air. *Philos. Trans. R. Soc. Math. Phys. Eng. Sci.* **2012**, *370* (1974), 4380–4403. https://doi.org/10.1098/rsta.2012.0137.

(11) Lees, E. W.; Goldman, M.; Fink, A. G.; Dvorak, D. J.; Salvatore, D. A.; Zhang, Z.; Loo, N. W. X.; Berlinguette, C. P. Electrodes Designed for Converting Bicarbonate into CO. *ACS Energy Lett.* **2020**, *5* (7), 2165–2173. https://doi.org/10.1021/acsenergylett.0c00898.

(12) Kim, Y.; Lees, E. W.; Donde, C.; Waizenegger, C. E. B.; Simpson, G. L.; Valji, A.; Berlinguette, C. P. Electrochemical Capture and Conversion of CO2 into Syngas. ChemRxiv (Chemical Engineering and Industrial Chemistry) August 29, 2023. https://doi.org/10.26434/chemrxiv-2023-hvjxn. (accessed 2024-04-22).

(13) Jackson, M. N.; Jung, O.; Lamotte, H. C.; Surendranath, Y. Donor-Dependent Promotion of Interfacial Proton-Coupled Electron Transfer in Aqueous Electrocatalysis. *ACS Catal.* **2019**, *9* (4), 3737–3743. https://doi.org/10.1021/acscatal.9b00056.

(14) Resasco, J.; Lum, Y.; Clark, E.; Zeledon, J. Z.; Bell, A. T. Effects of Anion Identity and Concentration on Electrochemical Reduction of CO2. *ChemElectroChem* **2018**, *5* (7), 1064–1072. https://doi.org/10.1002/celc.201701316.

(15) Marcandalli, G.; Boterman, K.; Koper, M. T. M. Understanding Hydrogen Evolution Reaction in Bicarbonate Buffer. *J. Catal.* **2022**, *405*, 346–354. https://doi.org/10.1016/j.jcat.2021.12.012.

(16) Marcandalli, G.; Goyal, A.; Koper, M. T. M. Electrolyte Effects on the Faradaic Efficiency of CO2 Reduction to CO on a Gold Electrode. *ACS Catal.* **2021**, *11* (9), 4936–4945. https://doi.org/10.1021/acscatal.1c00272.

(17) Yang, K.; Kas, R.; Smith, W. A. In Situ Infrared Spectroscopy Reveals Persistent Alkalinity near Electrode Surfaces during CO2 Electroreduction. *J. Am. Chem. Soc.* **2019**, *141* (40), 15891–15900. https://doi.org/10.1021/jacs.9b07000.

(18) Lees, E. W.; Bui, J. C.; Song, D.; Weber, A. Z.; Berlinguette, C. P. Continuum Model to Define the Chemistry and Mass Transfer in a Bicarbonate Electrolyzer. *ACS Energy Lett.* **2022**, *7* (2), 834–842. https://doi.org/10.1021/acsenergylett.1c02522.
